# Geometry-Based Bounds on the Capacity of Peak-Limited and Band-Limited Signals over the Additive White Gaussian Noise Channel at a High SNR

**DOI:** 10.3390/e27121192

**Published:** 2025-11-24

**Authors:** Michael Peleg, Shlomo Shamai

**Affiliations:** Faculty of Electrical and Computer Engineering, Technion-Israel Institute of Technology, Technion City, Haifa 32000, Israel

**Keywords:** peak power, amplitude, capacity, AWGN, band limited, entropy, upper bound

## Abstract

We present a new computable geometry-based upper bound on the capacity of peak-power-limited and band-limited signal over the Additive White Gaussian Noise Channel. The peak limit applies at continuous time. The bound is a function of the volume and shape of the transmitted signal set, namely the body, in the space of Nyquist-rate samples, comprising all of the points the transmitted signal can reach. At a high SNR, the bound is tight, better than previously known upper bounds and, together with a known lower bound, provides the capacity at an asymptotically high SNR. We found, using a numerical evaluation, the high-SNR capacity of signals with the structure used in Cyclic Prefix assisted Frequency Domain Equalization (CP-FDE) and OFDM for sequence length of up to 100 Nyquist intervals, and we present a conjecture that this result is correct for any sequence length and does not depend on the CPA-FDE structure. This paper extends the methodology developed in previous works. The penalty in power efficiency at a high SNR due to the peak power constraint relative to an average power constraint is about 7.5 dB in the low-pass case and about 5.4 dB in the band-pass case.

## 1. Introduction

We investigate the Peak-Power Limited and Band Limited (PPBL) Additive White Gaussian Noise (AWGN) channel in which the signal is band-limited and its instantaneous power cannot exceed the power *P*. Equivalently, the instantaneous amplitude cannot exceed $\sqrt{P}$. The peak power (peak amplitude) limit applies continuously, not only at the Nyquist-rate sampling times. This model is relevant to many systems in which the peak power is limited by the power amplifier at the transmitter. The model fits real system requirements even better since the introduction of Digital Pre-Distortion (DPD), e.g., [1,2], which linearizes the power amplifier up to its maximal transmit power, thus ensuring high and predictable performance with peak-power-limited signals. Clearly, the capacity limits of this channel are of major practical interest, e.g., the discussion of advances in new physical layer technologies in [3], the optimization in Section 3.6 of [2], and discussion in [4]. The importance of limiting the peak power is also reflected in many works analyzing and reducing the Peak to Average Power Ratio (PAPR), e.g., [5,6,7,8]. The impact of the peak power limit is classical in many communications settings since the beginning of the wireless communication era [9], and it is relevant to a variety of practical communication models, as, for example, fading channels and the like [4], and the optimization of constellations under the peak and average limits over the discrete-time channel [10]. The new results on the PPBL channel capacity may benefit recent and modern research on coding for this channel, e.g., [11], which investigates PAPR reduction for single carrier communication by transmit filter impulse-response optimization and [12], which induces a peak limit on 16-QAM Single-Carrier modulation by trellis shaping and exploits the shaping trellis as a component code of a turbo-coded scheme.

With the exact capacity of the classical Average-Power-Limited and Band-Limited (APBL) channel found by Shannon [9] and used widely for decades, the continuous-time PPBL channel capacity has only been studied sparsely, yielding lower and upper bounds on capacity with a wide gap in between. We believe the reason for this is the difficulty of analyzing this channel, as suggested already in [9]. Shannon analyzed this channel and presented lower and upper bounds on capacity in the low-pass case already in [9]. His lower bound was based on an achievability scheme with a low-pass signaling impulse response; his upper bound, valid at an asymptoticly high SNR, releases the peak limit to be applicable to Nyquist-rate samples only and then upper-bounds the entropy of the transmitted signals. The work by [13] used an improved impulse response to slightly improve the lower bound of [9] and provided an improved upper bound valid at all SNRs by releasing the peak limit to be applicable to Nyquist-rate samples only while applying the capacities of the corresponding sampled discrete-time channels available in [14,15]. It also extended the bounds to the more practical band-pass case. The capacity of the discrete-time peak-limited channel and analytical bounds on it are further investigated in [16,17]. Smith [14] derived the exact capacity-achieving distribution of signals over the discrete-time peak-limited channel and proved that it comprises discrete amplitudes. The capacity of this channel upper-bounds that of our continuously peak-limited channel, and both channels are peak-limited; still, the question whether the optimal signal in our case is similar to that in [14] remains unanswered. Recently, we presented a computable lower bound [18] on the capacity of the time-continuous PPBL channel at any Signal to Noise Ratio (SNR) applicable to the low-pass and the band-pass cases. The bound there was evaluated numerically over the Cyclic Prefix-assisted Frequency Domain Equalization (CP-FDE) or Orthogonal Frequency Division Multiplexing (OFDM) signaling. The CP-FDE signals are not strictly band-limited because they are limited in time; however, they are practically band-limited in the sense of zero inter-channel interference between users if the rules for cyclic prefix are adhered to, thus enabling the assignment of adjacent users to channels with no frequency gaps in between. This is applied, for example, in the multiuser uplink of the Long-Term Evolution (LTE) mobile communications system using Single-Carrier FDMA (SC-FDMA) [19].

The problem investigated here is related to communication over the Constrained Gaussian Channel (CGC) [20] in which a wideband peak-limited signal is fed into a transmit filter in the transmitter. As shown in [18], the capacity of the CGC is an upper bound of the capacity of the PPBL channel. The work by [20] uses the results of [21] on the Power Spectral Density (PSD) of unit processes, which are the inputs to the CGC channel, to derive the upper-bound capacity of the CGC channel, which is also valid for the PPBL channel. In [22,23], the approach is specified to the PPBL channel, gaining additional insights. The upper bound on the CCG channel was tightened in [23]. However, the CGC-based approach cannot provide an upper bound on capacity tight at high SNRs, as explained in Section 3.3.2 below. The review by [24] presents and categorizes a wide range of modulation schemes with different types of peak limits, including the CGC and the PPBL models.

Contributions: We derive a computable upper bound on the capacity of peak-limited CP-FDE and OFDM-type signals over the AWGN band-limited channel based on the volume and the shape of the signal set in the space of Nyquist-rate samples, comprising all of the points the transmitted signal can reach. This technique extends the geometric analysis of the transmitted signal set in [18] to analyze the geometric region into which the additive noise extends the transmit signal set to become the received signal set. The new upper bound is tighter at high SNRs than the known upper bounds. The existence of such a bound was mentioned in [18]; here, the bound is proved and its gap to the known lower bound is evaluated. The new upper bound approaches the lower bound at an asymptotically high SNR, thus providing capacity at an asymptotically high SNR. We also verify the lower bound in [18] by evaluating it using a more straightforward numerical method which enables us to control the standard deviation of the results and to present confidence intervals, unlike the more powerful but more complex method used in [18].

The methodology used in the current paper evolved relatively to that in the previous paper [18] as follows: In [18], we developed a lower bound on the capacity. The bound is a function of the volume of the transmitted signal set and uses the relation between maximal entropy and the volume of the signal set [9]. Since the signal set is *N*-dimensional, there was a need to develop a numerical technique to estimate the volume in the *N*-dimensional space, including a complex investigation of its accuracy. In the current paper, we developed an upper bound on the capacity to complement the lower bound [18]. To do so, we had to evaluate the entropy of the noisy received signal. Unlike the transmit signal [18], the received noisy signal is not confined to a bounded volume, and its entropy cannot be bounded by a function of the volume of the transmit signal set alone. Therefore, a new method bounding the contribution to the entropy of sections of space, each section extending over an unbounded volume, occupied by the received signal set, was developed. We utilized the volume-estimation core from [18] as an element of the new method.

Notation: Bold italic ***letters*** denote vectors. Log is the natural logarithm, unless stated otherwise. Differential entropy is denoted by *h*; *E* denotes the statistical expectation. The *N*-dimensional vector space of real variables is denoted *R^N^*. The Probability Density Function (PDF) of *x* is *p_x_*(*x*) or *p*(*x*). Convex bodies are denoted by calligraphic capital letters such as X. The unity-radius *N*-ball centered at 0 is B. The addition of convex bodies is performed by the Minkowski addition and is denoted by the usual +.

## 2. System Model

We begin with real-valued signals representing a low-pass channel. The system is presented in Figure 1. The encoder produces a real-valued low-pass signal *x*(*t*) in the frequency band |*f*| < *B*. The signal is constrained to be peak-limited, that is, $\left|x(t)\right|\leq \sqrt{P}$. The signal passes an AWGN channel and is decoded. The channel output *y*(*t*) is

(1) $$y\left(t\right)=x\left(t\right)+n\left(t\right)$$

where *n*(*t*) is a white Gaussian noise with power spectral density *N*_0_ (0.5*N*_0_ two-sided) and in-band power ${\sigma_{n}}^{2}=N_{0}B$. The Nyquist interval is *T = 0.5*/B. The channel and/or the decoder may include a brick-wall low-pass filter with bandwidth *B*; this does not influence the capacity, since *x*(*t*) is already band-limited. We seek the bounds on the capacity, which is the maximal Mutual Information (MUI) denoted *I*(*x*;*y*) per Nyquist interval. The Nyquist-rate-sampled *x*(*t*) is denoted by the vector ***x*** = (*x*_1_ *… x_k_ … x_N_*) of length *N, x_k_ = x*(*kT*). The vectors ***y*** and ***n*** denote the sampled received signal and noise, respectively. The sets of signals ***x*** and ***y*** are denoted X and Y, respectively.

The signal to noise ratio (SNR) is defined as $\rho =\frac{P}{{BN}_{0}}$, as in the classical APBL channel. The capacity in bits per Nyquist interval of an APBL channel is the following famous [9] equation:

(2) $$C_{a}=0.5\log_{2}\left(\frac{P}{N_{0}B}+1\right)$$

To render the numerical analysis feasible, *N* needed to be finite. To this end, we modelled *x*(*t*) as cyclic with a period of *NT* and then limited to a duration of *NT*. This is identical to the CP-FDE and OFDM signaling formats before adding the cyclic prefix, so our analysis is applicable to CP-FDE and OFDM. In this setting, *x*(*t*) can be expressed in terms of its Nyquist-rate samples, e.g., [25], as

(3) $$x\left(t\right)=\sum_{i=0}^{N-1}x_{i}\cdot \varphi \left(t-iT\right);\;0\leq t<NT$$

where *φ*(*t*) is the periodic sinc function (4), also known as the Dirichlet kernel, instead of the usual sinc function (see, e.g., [25]):

(4) $$\varphi \left(t\right)=\frac{sin(\pi t/T)}{N\cdot sin\left[\pi t/(N\cdot T)\right]}$$

The expression is valid for odd *N* and has a counterpart for even *N* (see, e.g., [25]). Note that $\varphi \left(t\right)$ is periodic, with a period of *NT*. The functions $\varphi \left(t-iT\right),\;i=0\ldots N-1$ over 0 < *t* < *NT* form an orthogonal basis of our band-limited periodic signals, as can be shown easily using Equation (11) in [25].

The definition of capacity, which requires long sequences, is applicable if each error-correction block comprises many *N*-samples long CP-FDE signal segments (3), with each segment different and generated jointly by the error-correcting encoder. In the case of CP-FDE and OFDM signaling, each signal segment (3) of duration *NT* is to be prepended by its cyclic prefix and concatenated with the other segments to form the error-correction block. Thus, our model directly applies to CP-FDE and OFDM.

If *N* is increased, the cyclic prefixes become negligible and the signal with large and finite *N* is similar to a standard low-pass signal by the similarity of the sinc and periodic sinc (4) impulse responses, as follows: The standard low-pass signal *x*(*t*) is determined at time *t* by its Nyquist-rate samples *x_i_* weighted by the sinc impulse response. We shall partition those samples into two groups: The first group comprises the *N*-neighboring samples *x_i_* centered around *t*, such that |*iT-t*| < *NT*/2. Each such sample has one parallel sample in the finite *N* case (3), weighted by the periodic sinc impulse response (4). Comparison of the two impulse responses shows that they are similar at the relevant times; the differences are smaller than approximately $\frac{\pi -2}{\pi N}$ relative to the peak of the impulse response, which is 0.0071 for *N* = 51 and smaller for larger *N*. The second group comprises all the other more distant samples. These have no parallel in the finite *N* case; however, the sinc impulse response relevant to those samples is low, with a maxima of about 2 / (π*N*) relative to the peak of the impulse response; thus, their influence decreases with growing *N*.

## 3. Bounds on Capacity

### 3.1. On the Shape of the Signal Set

The transmit signal set X of vectors ***x*** of Nyquist-rate samples has the following attributes, illustrated in Figure 2: The set X is convex, that is, if signals $x_{1}$ and $x_{2}$ are in the signal set, so is $ax_{1}+(1-a)x_{2}$ for any real positive scalar *a <* 1 (see [18] for proof and discussion). The signal region is symmetric, that is, if ***x*** is permissible, then −***x*** is too. The set X is time-invariant, that is, invariant with respect to a time-shift in an integer number of Nyquist-rate samples; the shift becomes circular in the case of the CP-FDE channel model. The set X is confined to be in an *N*-cube, defined by $\left|x_{i}\right|\leq \sqrt{P}$ by the peak limit when applied to the samples. The set X touches the *N*-cube at the center of each of its faces at the points $x=\ldots \;0,\;0,\;0\;\mp \sqrt{P\;}\;,\;0,\;0,\;\ldots \;$. Thus, all the faces of the hypercube are supporting hyperplanes of X. There are many points of X on each such supporting hyperplane, since there are many different band-limited signals attaining maximal absolute value at any *t = t_m_*. The set X does not reach most of the *N*-cube vertexes (corners); however, it does touch the special vertexes $x=\mp \sqrt{P\;}[1,\;1,\;\ldots 1]$, which produce a constant $x\left(t\right)=\pm \sqrt{P}$. The distance from the origin to any vertex of the *N*-cube is larger by a factor of $\sqrt{N\;}$ relative to the distance from the origin to the centers of the *N*-cube faces. Using (3) and (4) and shifting the Nyquist-rate sampling times by a fraction of the sampling interval from *kT*, *k* = 1…*N*, to *kT* + τ transforms x from one set of orthogonal coordinates $\varphi \left(t-iT\right)$ to another $\varphi \left(t-\tau -iT\right)$ and creates an additional hypercube, confining X and having the same properties of the faces.

### 3.2. Volume-Based Lower Bound

The geometrical lower bound on the capacity *C*, based on the Entropy Power Inequality (EPI), was presented in [18]. We summarize the relevant expressions below.

(5) $$\;C\geq 0.5\log_{2}\left(\frac{\gamma \cdot P}{N_{0}B}+1\right)=0.5\log_{2}\left(\gamma \cdot \rho +1\right)$$

The power factor γ≤1 is

(6) $$\gamma =\frac{V_{x}^{\frac{2}{N}}}{2\pi e}$$

where *V_x_* is the volume of the signal set X evaluated for signals with power *P* = 1.

**Figure 3:**
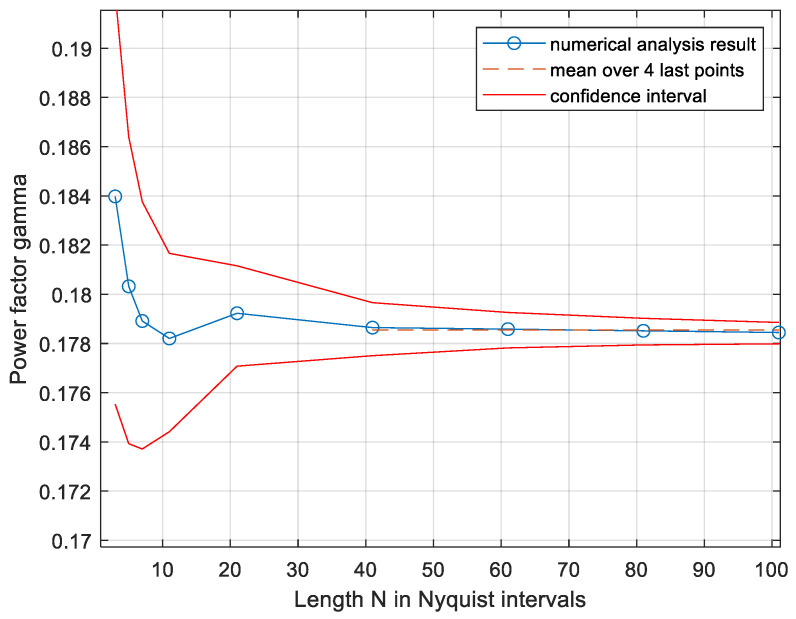
Lower bound on the power factor as a function of the sequence length. The red lines are the confidence interval $\mp 4\sigma_{p}$. The number of simulations *N_t_* was increased with increasing *N* to yield about 1000 hits inside the signal set.

**Figure 1:**
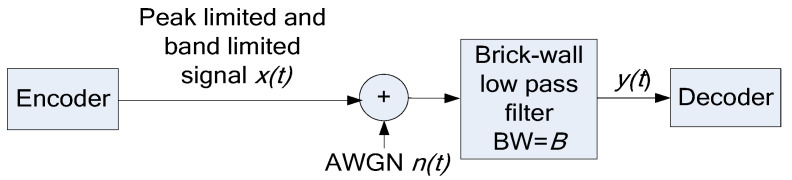
System model of communication over the PPBL channel, low-pass case.

An updated numerical method with an accuracy analysis added is presented next. The accuracy analysis is independent of the shape of X, rendering it vastly simpler than its counterpart in [18]. As stated above, all the vectors ***x*** of samples comprising X are in an *N*-cube. To evaluate *V_x_*, the components of ***x*** are generated at random u.i.i.d in $-\sqrt{P}\leq x_{i}\leq \sqrt{P}$. This covers the volume of the hypercube uniformly and is repeated *N_t_* times. Each ***x*** is tested if being in X, that is, not violating the peak limit in the transmission at any time *t*. Then *V_x_* is the probability *p_c_* of ***x*** being inside X multiplied by the volume ${\left(2\sqrt{P}\right)}^{N}$ of the hypercube, yielding

(7) $$V_{x}=p_{c}\cdot {\left(2\sqrt{P}\right)}^{N}$$

and, from (6), the power factor, evaluated without loss of generality at *P* = 1, is

(8) $$\gamma =\frac{{2p_{c}}^{2/N}}{\pi e}$$

next, from (5)

(9) $$C\geq 0.5\log\left(\frac{P}{{\sigma_{n}}^{2}}\cdot \frac{2{p_{c}}^{2/N}}{\pi e}+1\right),$$

thus, the power factor is a product of $\frac{2}{\pi e}$ present in the asymptotic high SNR upper bound [9] based on the discrete-time channel and of the new term ${p_{c}}^{2/N}$, accounting for the peak constraint applied also to all times between the Nyquist-rate samples. This is a somewhat different method of evaluating the power factor γ relative to the method used in [18], so it is a partial verification. Furthermore, using this new and simpler method, the accuracy of the numerical evaluation can be controlled better, showing that the results in Figure 3 are accurate, as follows: The value of *p_c_* is evaluated by generating *x*(*t*) at random for *N_t_* times. The estimated *p_c_* is a binomial random variable, scaled by 1/*N*_t_, of mean *p_c_* and standard deviation of

(10) $$\sigma_{p}=\sqrt{p_{c}(1-p_{c})/N_{t}}$$

To plot a confidence interval of the lower bound (9), we can plot the bound along with lines offsetting *p_c_* by $\mp 4\sigma_{p}$. This is shown in Figure 3 using an oversampling factor *N_k_* = 30.

Thus, the power factor is 0.1785 and is very stable with respect to increasing *N*. This is the same value as the one derived in Figure 12 of [18] by a different numerical method. The stability with rising *N*, the narrow confidence interval of less than 1% of the power factor itself, and the similarity, at sufficiently large *N,* of the standard low-pass signals to the CP-FDE signals used in the analysis (presented at the end of the system model section) lead to the following conjecture:

**Conjecture** **1.** *The result γ = 0.1785 is also valid for infinite N and for general low-pass signals that are not necessarily a CP-FDE type.*

### 3.3. Geometry-Based Upper Bound

#### 3.3.1. Analysis

The capacity per sample is

(11) $$C=\frac{1}{N}\left[h\left(y\right)-h\left(y|x\right)\right]=\frac{1}{N}\left[h\left(y\right)-h\left(n\right)\right]=\frac{1}{N}h\left(y\right)-0.5log(2\pi e{\sigma_{n}}^{2})$$

maximized over the PDF of the transmit signals ***x***. An interesting progress on a geometry-based upper bound over a discrete-time MIMO channel was achieved in [26]. One of its results, section *Asymptotic gap* there on the tightness of the EPI-based lower bound, applies to our problem (explanation: Equation (7) of [26] is the parallel of our (1) in its vector form with a finite *N* provided ***H*** =***I** _NxN_*, and our problem conforms to the convexity constraint on the signal set X as required in [26]). Thus, at an asymptotically high SNR, the lower bound (5), based on EPI, is tight. However, for the continuous-time channel here, we could not derive the intrinsic volumes of X required in the Steiner formula as performed in discrete-time in [26], so we are not able to derive quantitative results based on those, e.g., how high the SNR needs to be for the upper bound to be tight. To achieve this, we used a different approach. We proceeded in two stages: First, we established the following Theorem 2. Then we developed a numerical procedure upper-bounding the volume *V*_w_ in (12) and upper bounding the capacity (11) using (12).

**Theorem** **2.** *The entropy of the received signal is upper bounded as follows*

(12) $$h\left(y\right)\leq log(V_{w})$$

*where V_w_ is volume of W and W is the Minkowski sum *

(13) $$W=X+R_{f}\cdot B,$$

*B denotes an N-ball of unit radius and*$R_{f}=\sigma_{n}\cdot \sqrt{2e}\;{\left[\Gamma \left(\frac{N}{2}+1\right)\right]}^{\frac{1}{N}},\;which\;is\;approximately$ $R_{f}\approx \sigma_{n}\cdot \sqrt{N}$.

Remark: The Minkowski sums, similar to (13), of signal sets and noise sets appear in previous works, e.g., [26] and the references within, usually with infinite *N*, leading to the “ball-hardening” of the noise. In our case, with a finite *N*, the received signal cannot be confined to a finite volume (see Figure 4) and the object B in (13) is not a noise set, but rather a body, illustrated in Figure 5, the addition of which to X ensures the bound(12); see the following proof:

**Proof of Theorem** **2.** We start with the attributes of the PDF *p_y_*(***y***) of the received signal at the region outside of X. For a pair ***x, y*** the corresponding noise is ***n*(*x, y*)** =***y* − *x*** with *N*-dimensional normal distribution and variance per dimension of *σ*^2^. Then

(14) $$p(y)=\int_{all\;x\;}p\left(x\right)p(y|x)dx=\int_{allx\;}p\left(x\right)p\left[n\left(x,y\right)\right]dx$$

Now, let us introduce a supporting (tangent) hyperplane touching the convex body X at a point ***z*** on it (see Figure 4). Line *G,* starting at ***z***, is perpendicular to the supporting hyperplane. Consider a point ***y*** on *G*.

Note that the supporting hyperplane is *N*-1 dimensional and the distance *g* from it to the point ***y*** is one-dimensional. Note that, for any ***z*** on the surface of X, there is at least one supporting hyperplane containing it and the associated perpendicular line marked *G* in Figure 4. By the normal distribution of ***n***,

$$p\left[n\left(y,x\right)\right]=C_{1}(x)\;e^{-0.5{\left(\left(g+C_{2}(x)\right)/\sigma_{n}\right)}^{2}}$$

for some pair of positive scalars $C_{1}\left(x\right),\;C_{2}\left(x\right)$. Then, following (14), the slowest decaying *p_y_*(***y***) with respect to *g*, used in the proof of Proposition 3 below, is

(15) $${p_{y}^{s}(y)=C_{4}\cdot e}^{-0.5{\left(g/\sigma_{n}\right)}^{2}}$$

for some constant *C_4_*. The slowest decaying $p_{y}^{s}(y)$ denotes that, for any $p_{y}(y)$, the ratio $p_{y}(y)/p_{y}^{s}(y)$ is non-increasing in g. Denote ${p_{y}^{s}(g)=C_{4}\cdot e}^{-0.5{\left(g/\sigma_{n}\right)}^{2}}$.

Next, we partition the volume of the received signals ***y*** outside of X into sections constructed as follows:

1. The base of each section is a small area on the *N*-1 -dimensional surface of X around some point denoted ***z***. or a small *M*-1—dimensional body, *M < N*, around ***z*** in the surface of X or the point ***z***itself.
2. The section extends from the base outward along a line *G* perpendicular to the supporting hyperplane at ***z***. The section is narrow enough to render *p_y_*(***y***) a function *p_y_*(*g*) of *g* only where *g* is the distance of ***y*** in the section from the supporting hyperplane. There may be several supporting hyperplanes at any ***z***, e.g., at the special vertexes presented in Section 3.1 above.
3. The intersection between the section and a hyperplane parallel to the supporting hyperplane and at a distance *g* from it has an (*N*-1) volume *S*(*g*). The volume *S*(*g*) is a non-decreasing function of *g*.

Figure 5 illustrates examples of such sections. A section, a base of which is on a flat part of the surface of X, may be a cylinder with a constant *S*(*g*). A section the base of which is the vertex of the *N*-cube will include cones (single-nappe infinitely extending cones, that is, corresponding to values of *g* from zero to infinity), with *S*(*g*) proportional to

(16) $${S(g)=C_{3}\cdot g}^{N-1}.$$

The growth of *S*(*g*) in (16) is the fastest needed one, since such sections can cover an *N*-ball from a single center point ***z***; see also the relation to the Steiner formula below.

Each such section extends to infinity, that is, *g* extends from 0 to infinity. We want to calculate the entropy contributed to *h*(***y***) by a single section, indexed *i*. This contribution to entropy is the *N*-dimensional integral

$$h_{i}=-\int_{section\;i}p_{y}(y)\log\left(p_{y}\left(y\right)\right)dy$$

Denote the probability of ***y*** to be in a section *i* as *p_i_*. The volume of each section can be integrated in slices taken at each value of *g*, the volume of each slice being dg·S(g); the volume of the section is

(17) $$V_{i}=\int_{0}^{\infty }S(g)dg$$

which is infinite, and

(18) $$h_{i}=\int_{0}^{\infty }p_{y}(g)S(g)\log\frac{1}{p_{y}\left(g\right)}dg$$

and

(19) $$p_{i}=\int_{0}^{\infty }p_{y}(g)S(g)dg$$

Note that, by(15), *p_y_*(*g*) is a decreasing function of *g* and *S*(*g*) is an increasing one by the definition of the sections construction above. Let us consider a situation in which *p_i_* is fixed. Then the following proposition holds:

**Proposition** **3.** *The p_y_*(*g*) *which maximizes h_i_ for a given p_i_ and S*(*g*) *is the slowest decreasing one *$p_{y}^{s}\left(g\right)\;in$* (15).*

**Proof.**  Let there be two different functions *p_y_*(*g*)*,* $p_{y}^{s}(g)$*,* both upholding (19). Such two functions must have crossings due to (19) and due to identical *p_i_*, and there can be only one such crossing due to $p_{y}^{s}\left(g\right)$ being the slowest decreasing. The function $p_{y}^{s}\left(g\right)$ can be obtained from *p_y_*(*g*) by incremental steps described below, each step moving some of the function mass from lower to higher region of *g*. This modification will enlarge *h_i_*, since this operation moves probability mass from regions with lower values of $\log\frac{1}{p_{y}\left(g\right)}$ to regions with higher values (see (18)). Each step transforms *p_y_*_1_(*g*) into *p_y_*_2_(*g*) so that at the first step *p_y_*_1_(*g*) = *p_y_* (*g*) and at the last step $p_{y2}\left(g\right)=p_{y}^{s}\left(g\right)$. At each step, *p_y_*_1_(*g*) is reduced in a narrow region *dg* of *g* around *g*_1_ and increased around a narrow region of *g* around *g*_2_, such that *g*_1_*_<_ g*_2_, adjusting the increment to keep *p_i_* in (19) constant while accounting for *S*(*g*). Thus, to produce *p_y_*_2_(*g*), *p_y_*_1_(*g*_1_) will be decremented by some *dp* and *p_y_*_1_(*g*_2_) will be incremented by $dp\frac{S(g_{1})}{S(g_{2})}$. Define *p_y_*_3_(*g*) as similar to *p_y_*_2_(*g*), except that *dp* is increased so as to yield *p_y_*_3_(*g*_1_) = *p_y_*_3_(*g*_2_). Clearly, *h_i_*, associated with *p_y_*_3_, will be at least as large as that associated with *p_y_*_1_, since uniform distribution maximizes entropy. Since *p_y_*_2_(*g*) is a linear combination of *p_y_*_1_(*g*) and of *p_y_*_3_(*g*), the transition from *p_y_*_1_ to *p_y_*_2_ increases *h_i_* in (18) by the concavity of the entropy function. □

So, inserting(15) and (16) into (18) will yield the upper bound on the entropy in a section with *S*(*g*) as given in(16). Consider evaluating (18) after such an insertion parametrized by *p_i_* = 1 and by *S*(*g*) being the surface of an *N*-ball of radius *g*. In this case, *p**_y_***(*g*) is the PDF of the *N*-dimensional Gaussian noise; thus, *h_i_* will be equal to the corresponding entropy, which is

$$h_{n}=\frac{N}{2}log(2\pi e{\sigma_{n}}^{2}).$$

Now let us evaluate (18) under the same conditions and using the same *p**_y_***(*g*), except for setting an arbitrary *p_i_* and with *S*(*g*) reduced by multiplying it by a small factor α. This yields a narrow conical section. The changes will scale *p**_y_***(*g*) by $\frac{p_{i}}{\alpha }$. Then (18) yields

$$h_{i}=\int_{0}^{\infty }{\frac{p_{i}}{\alpha }p}_{y}(g)\alpha S(g)\log\frac{\alpha }{{p_{i}\cdot p}_{y}\left(g\right)}dg$$

$$h_{i}=p_{i}\int_{0}^{\infty }p_{y}(g)S(g)\log\frac{1}{p_{y}\left(g\right)}dg+p_{i}\log\frac{\alpha }{p_{i}}\int_{0}^{\infty }p_{y}(g)S(g)dg$$

(20) $$h_{i}=p_{i}h_{n}+p_{i}\log\frac{\alpha }{p_{i}}$$

For the sake of upper-bounding *h*(***y***), we shall replace each section *i* by an equivalent section having the same $h_{i}$, the same probability *p_i_*, and the same shape *S_i_*(*g*) as the original section, but limited to the region *0 < g* < *R_f_*. The volume of the equivalent section is denoted $V_{i}^{\prime }$. The vector ***y*** occurs in the equivalent section with probability *p_i_* with the uniform PDF $p_{i}\left(y\right)=p_{i}/V_{i}^{\prime }$*. R_f_* is set so that *h_i_* is preserved. The equivalence of *p_i_* and *h_i_* in each section renders the global new PDF legitimate. The new PDF has support of a limited volume *V_W_*, the logarithm of which is an upper bound on entropy, and (12) follows. The contribution of the equivalent section to entropy is

(21) $$h_{i}=-p_{i}\log(p_{i}/V_{i}^{\prime })=p_{i}log(V_{i}^{\prime })-p_{i}log(p_{i})$$

Let $V_{i}^{\prime }$ fit into the conical section (16) above up to a radius *R_f_*. Then

$$V_{i}^{\prime }={\alpha V}_{B}{R_{f}}^{N}$$

where *V_B_* is the volume of a unity radius *N*-ball and (21) becomes

$$h_{i}=p_{i}log(V_{B}{R_{f}}^{N})+p_{i}\log\frac{\alpha }{p_{i}}$$

So, to upper bound the section entropy (20) by that of the uniformly distributed $V_{i}^{\prime }$ we need

(22) $$\log\left(V_{B}{R_{f}}^{N}\right){=h}_{i}{=h}_{n}$$

and this is independent of α and *p_i_*. As presented in [9] and later termed the ‘noise ball hardening effect’, this yields, for large *N*, the radius of the noise sphere of

$$R_{f}\approx \sigma_{n}\sqrt{N}.$$

This is the approximate value of *R_f_* stated in Theorem 2, and is easily verified using Stirling’s formula, as presented in [27]. So, placing a cone limited to radius *R_f_* with the signal ***y*** distributed uniformly within provides at least the same entropy gain *h_i_* as the original cone with unlimited *g*; thus, the conical sections which extend to infinity in Figure 5 can be replaced by the same conical sections, but with limited radius *R_f_*, as illustrated by the dashed line in Figure 5 and with uniform pdf of ***y*** in each section. The exact evaluation of *R_f_* from (22), without the large *N* approximation, starts with the exact volume of an *N*-ball with a unity radius (e.g., [27]):

$$V_{B}=\frac{\pi^{N/2}}{\Gamma (\frac{N}{2}+1)}$$

from (22)

$$\log\left(\frac{\pi^{N/2}}{\Gamma (\frac{N}{2}+1)}{R_{f}}^{N}\right)=N\log\left(\sigma_{n}\cdot \sqrt{2\pi e}\right)$$

$${R_{f}}^{N}=\frac{\left(\sigma_{n}\cdot \sqrt{2\pi e}\right)N}{\pi^{N/2}}\Gamma (\frac{N}{2}+1)$$

(23) $$\;R_{f}=\sigma_{n}\cdot \sqrt{2e}\;{\left[\Gamma \left(\frac{N}{2}+1\right)\right]}^{1/N}$$

as stated in the theorem. The other extreme case is the cylindrical section, that is, constant *S*(*g*) = S_0_; we shall have from (15) and (19)

$$p_{y}\left(g\right)=\frac{2p_{i}}{S_{0}}p_{G}(g)$$

where $p_{G}(g)\;$ is the PDF of the normal variable with zero mean and standard deviation of *σ_n_*. Then, from (18),

$$h_{i}=S_{0}\int_{0}^{\infty }\frac{2p_{i}}{S_{0}}p_{G}(g)\log\frac{1}{\frac{2p_{i}}{S_{0}}p_{G}(g)}dg=p_{i}\log\left(\sigma_{n}\cdot \sqrt{2\pi e}\right)+p_{i}\log\left(\frac{S_{0}}{2p_{i}}\right)$$

while the volume of a cylinder of length *R_f_* is

$$V_{i}=S_{0}R_{f}.$$

Then, equating the last $h_{i}$ with the entropy (21) of ***y*** distributed uniformly in *V_i_*,

$$p_{i}\log\left(\sigma_{n}\cdot \sqrt{2\pi e}\right)+p_{i}\log\left(\frac{S_{0}}{2p_{i}}\right)=p_{i}log(S_{0}R_{f}))-p_{i}log(p_{i})$$

(24) $$R_{f}=\sigma_{n}\cdot \sqrt{\frac{\pi e}{2}}$$

which is much smaller than for the conical section. Another trackable section type is a cone-like shape with ${S(g)=C_{5}\cdot g}^{M-1},\;2\leq M<N$ and with a volume being *M*-th power of *R_f_* by (17). Its radius *R_f_* is readily derived as in (23) with *N* replaced by *M*; such a radius is smaller than *R_f_* in (23). Indeed, inserting *N* = 1 into (23) yields(24), as expected, considering that (16) with unity *N* yields the cylindrical shape. The combination of the conical and cylindrical sections above yields an equation of the volume of the part external to X of X + *R*B*_N_* for any positive *R* since, using the Steiner formula (e.g., [28]), such volumes are polynomials of degree *N* in *R*. It is possible that additional shape types are needed for the partition of the whole volume into the sections; if so, arbitrary additional types conforming to the sections description above can be formed with no increase in *R_f_*, as presented in Appendix A. End of proof of Theorem 2. □

Theorem 2 has some similarity to section V in [26]; the main difference is that Theorem 2 does not involve the limit of an infinite number of channel uses. In principle, the radius *R_f_* can be reduced relative to (23) according to the local curvature of the surface of X (see Figure 5). However, to control the complexity and reliability of the numerical procedures, we shall use the maximal radius (23) all over, this will render the bound less tight. Equivalently, using the ball of the radius given by (23) is a tight bound around the vertexes and it exaggerates the entropy in the vicinity of flat faces, and the Minkowski addition of a ball in Theorem 2 overestimates the capacity.

Note that, at an infinite SNR, the set W is identical to X, as evident from the vanishing *R_f_* in (23), thus,, the upper bound becomes equal to the lower bound (5), regardless of the shape of X, as predicted also by [26].

#### 3.3.2. Numerical Analysis of the Upper Bound

**Figure 2:**
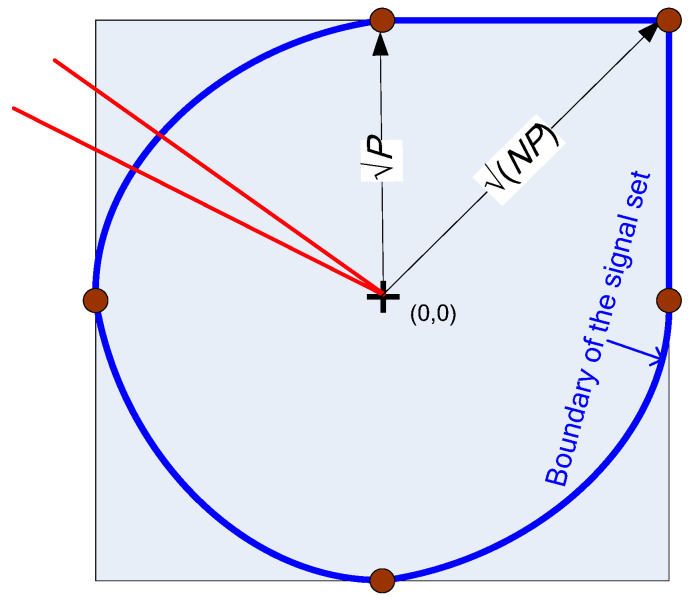
Illustration of the shape of the signal set. The square represents the N-cube. The red lines represent the cone used in Section 3.3.2.

**Figure 4:**
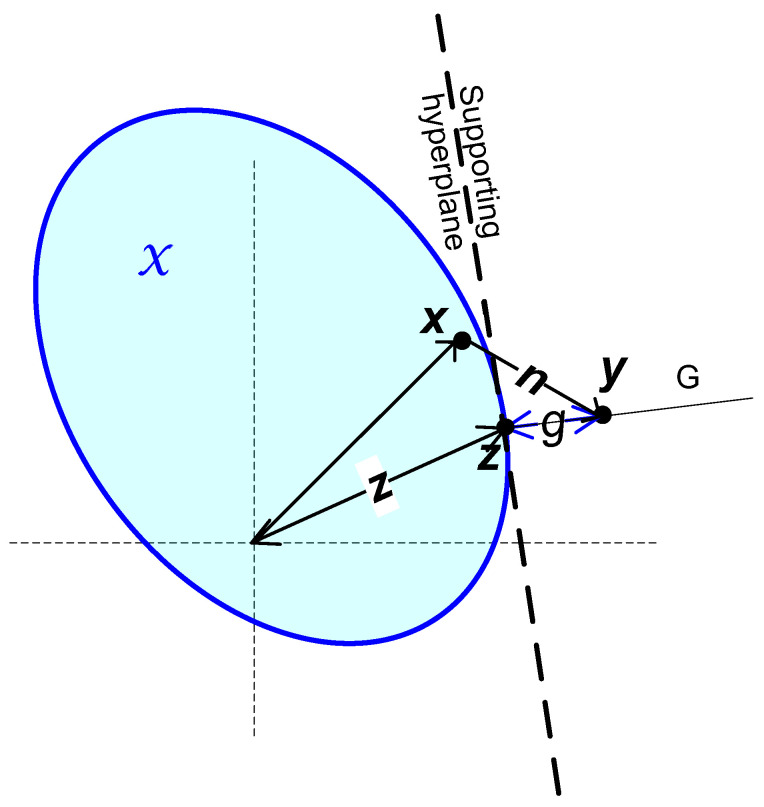
The supporting hyperplane, the transmitted signal ***x*** in the set X, the noise ***n***, the received signal ***y*** and the line *G*.

We need to upper bound the volume *V_w_* of W to upper-bound the entropy (12) and then the capacity(11). Since we do not have a simple expression of the shape of X (see Figure 2), we need a numerical evaluation, as presented next.

The numerical evaluation of *V_w_* is based on the method used to evaluate *V_X_* presented in [18] with an extension. As in [18], we partition the space of the signals ***y*** and ***x*** into narrow conical sections (*N*-cones) with apexes at the origin, as illustrated by the red lines in Figure 2 and Figure 6. The conical sections are visited at random by generating random *x*(*t*), as in [18]; each *x*(*t*) is scaled so that $\max_{t}\left|x\left(t\right)\right|=\sqrt{P}$ so the vector ***x*** of samples is on the surface of X. Then, we evaluate *r* = |***x***|, the local radius of X, that is, the distance from the origin to the point ***x*** on the surface of X. The volume *V_x_* in [18] is evaluated from many values of *r*. Here, instead of using *r* directly to evaluate *V_x_*, as in [18], we use *r_w_,* which is the distance from the origin to the surface of W, to evaluate *V_w_*. The radius *r_w_* is upper bounded as

(25) $$r_{w}=r\frac{\sqrt{P}+R_{f}}{\sqrt{P}}$$

justified as follows: At some *t* = *t_m_*, we have $\left|x\left(t_{m}\right)\right|=\sqrt{P}$. We represent *x*(*t*) in its vector form by the sequence of the Nyquist-rate samples at times *kT* + *τ* shifted by 0< τ < *T*, such that *t_m_* is one of the sampling times. Then, as explained in Section 3.1 above, ***x*** is a point on a supporting hyperplane of X. Next, we upscale ***x*** to produce the point $y=x\frac{\sqrt{P}+R_{f}}{\sqrt{P}}$. Clearly $\left|y\left(t_{m}\right)\right|=\sqrt{P}+R_{f}$; thus, ***y*** is at a distance of at least *R_f_* from ***x***. And this also holds for all other signals *x*(*t*) due to the peak power constraint at time *t_m_*. Then, by the Minkowski sum (13), ***y*** is on the surface of W or outside W. Since *r* scales identically to *x*(*t*), *r_w_* in (25) is guaranteed to be a local radius reaching the surface of W or beyond.

Additionally to (25), *r_w_* is limited at each trial not to exceed an *N*-ball with a radius of ${\sqrt{PN}+R}_{f}$. The evaluation of *V_W_* is performed as the evaluation of *V_x_* in [18] including the importance sampling while *r* in [18] is replaced by *r_w_*(25).

The results are presented in Figure 7 compared to the EPI-based lower bound in (5) and (9), to the average power-limited capacity (2), and to the discrete-time upper bound using Equation (1) in Kellips [16]. We also compared to the unit process upper bound [23], which is equivalent to a power loss by a ratio of 0.9259 relative to (2), which might be improved, but not below a ratio of 0.5 due to lemma 3 in [22]; thus, the CGC-based approach cannot provide an upper bound on a capacity that is tight at a high SNR.

**Figure 5:**
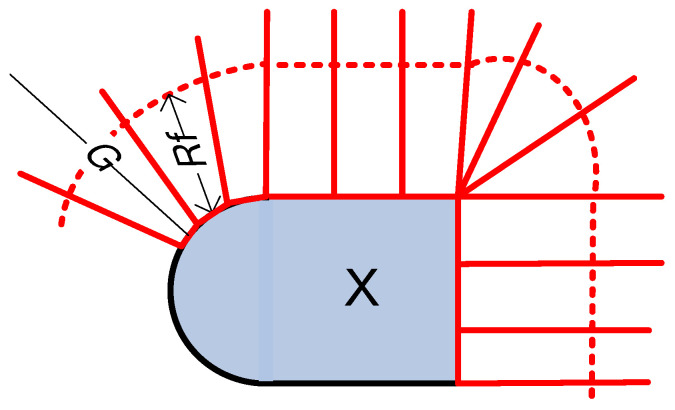
The gray signal set X and the red sections surrounding it. The dashed line illustrates the boundaries of the entropy-equivalent sections.

**Figure 6:**
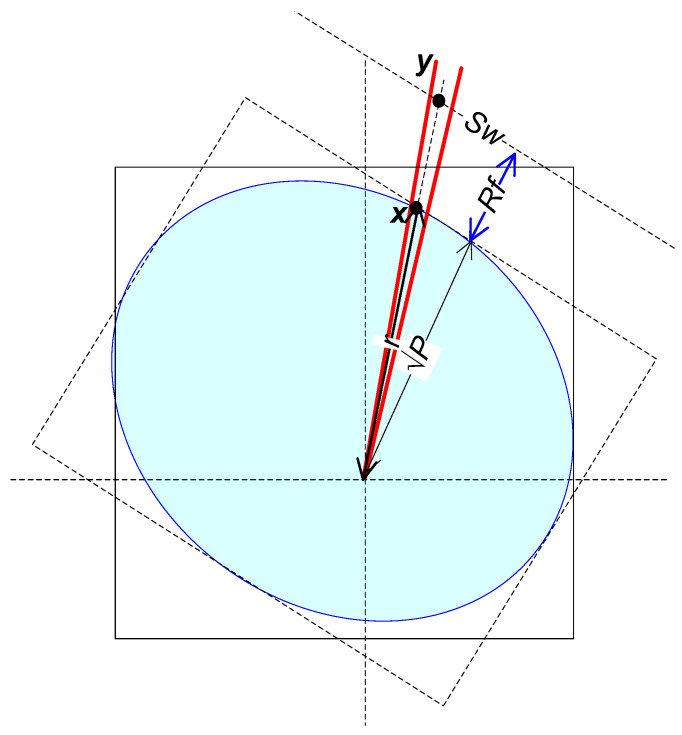
Elements of the upper-bound numerical analysis. The light blue ellipsoid is an illustration of X. The red lines illustrate the *N*-cone. The full-line square represents the hypercube confining X. The dashed square illustrates the hypercube formed by time-shifted Nyquist-rate samples. *S_w_* is the surface of W.

**Figure 7:**
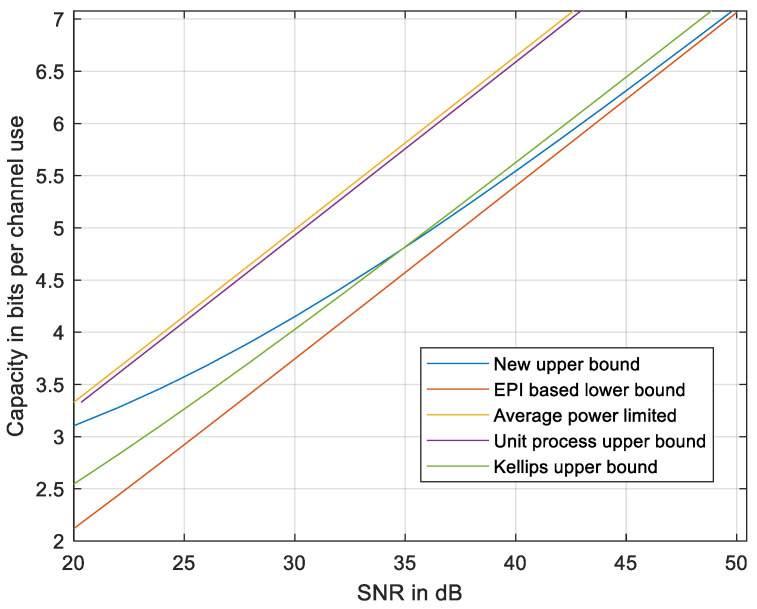
The new upper bound compared to the EPI-based lower bound [18], to the average power-limited capacity, to the unit process upper bound [23], and to the discrete-time upper bound using Kellips [16]. *N* = 101.

**Figure 8:**
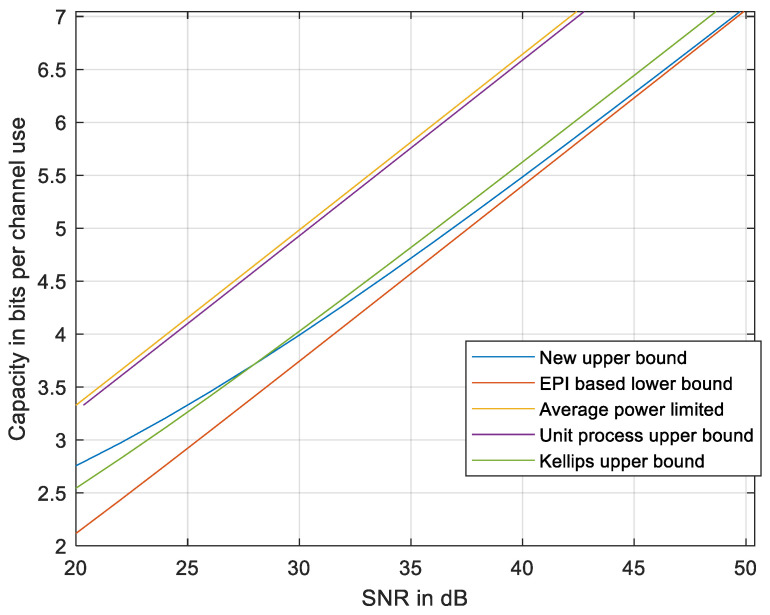
The new upper bound compared to the EPI-based lower bound [18], to the average power-limited capacity, to the unit process upper bound [21], and to the discrete-time upper bound using Kellips [16]. *N* = 31.

The gap between the upper and the lower bounds at an intermediate SNR is wider at high *N* and decreases at lower *N* as seen in Figure 8 with *N* = 31. Thus, the upper bound may be less tight at high *N*. Further improvement of the upper bound might be addressed in a future work that would refine the inequalities used in developing the upper bound, e.g., adapting *R_f_* to the local curvature of the signal set X and evaluating *h_i_* in each section, depicted in Figure 5, by a new method yet to be developed.

**Figure 9:**
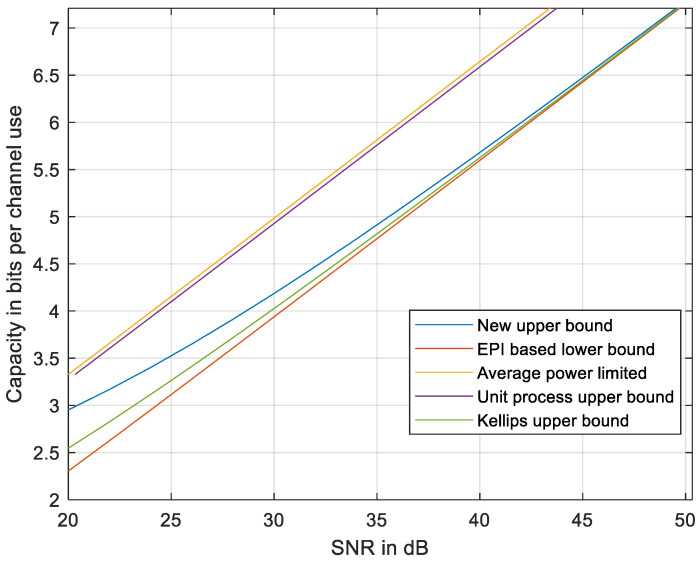
The bounds if the peak limit on *x*(*t*) is applied at the sampling times *kT* only. *N* = 31.

To verify the method and the software, we applied the peak limit on the Nyquist samples only. The results (see Figure 9) were expected to match the works on peak-limited discrete-time channels in [14,16,17], as presented in the Introduction. Indeed, the new upper bound and the upper bound in [16] on discrete-time channels plotted in Figure 9 do match at a high SNR while showing that, in this simple case, the tight upper bound in Kellips [16] is superior to ours at low and intermediate SNRs.

## 4. Band-Pass Signals

### 4.1. Signal Representation

The analysis is readily extended to band-pass signals represented in the complex-valued baseband. We reuse the previous notation with modifications, as follows. The encoder produces a complex-valued low-pass signal *x*(*t*) in the frequencies |*f*| < 0.5*B*. The noise is complex-valued with a power spectral density *N*_0_ (two-sided). The signal is peak-limited, that is, $\left|x\left(t\right)\right|\leq \sqrt{P}\;for\;all\;t\;$. The signal to noise ratio is defined as $\rho =\frac{P}{{BN}_{0}}$. The classical capacity per Nyquist-rate sample of the APL channel is

(26) $$C_{a}=\log_{2}\left(\frac{P}{N_{0}B}+1\right)$$

The real and imaginary components of the noise have a power of $\sigma_{n}^{2}$/2 = *N*_0_*B*/2 each, and the per-symbol noise entropy is $h\left(n\right)=\log\left(\sigma_{n}^{2}\cdot \pi e\right)$. The complex samples are represented by pairs of real numbers, and the volume *V_x_* is computed in an 2*N*-dimensional real-valued space. The *N*-cubes containing X in the low-pass case, which can be considered Cartesian products of line segments each representing one peak-limited real-valued sample, are replaced by Cartesian products of *N* disks with a radius of $\sqrt{P}$, each representing one complex sample peak-limited to $\sqrt{P}$. Denote the set defined by this product for *P* = 1 by D.

### 4.2. Lower Bound

The power factor and the EPI-based lower bound (see [18]) are now

(27) $$\gamma =\frac{1}{\pi e}V_{x}^{\frac{1}{N}}$$

(28) $$C\geq \log_{2}\left(\frac{\gamma \cdot P}{N_{0}B}+1\right)$$

**Figure 10:**
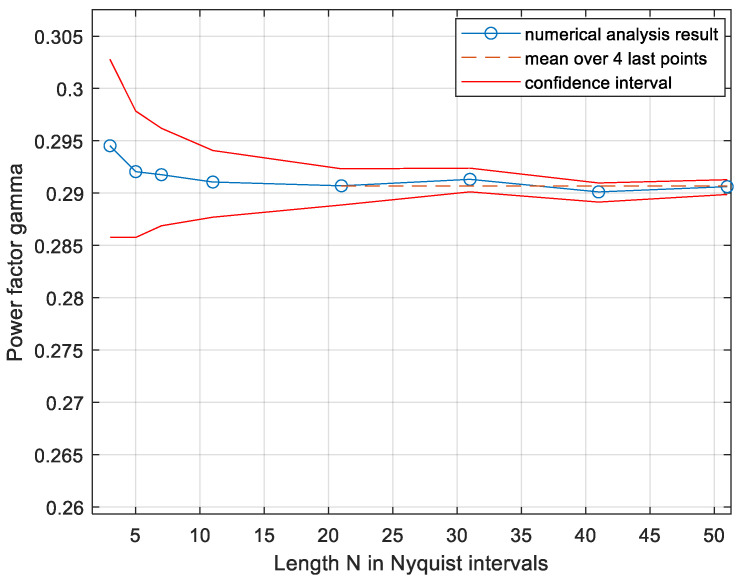
Lower bound on the power factor in the band-pass channel as a function of the sequence length. The red lines are the confidence interval $\mp 4\sigma_{p}$. The numbers of simulations *N*_t_ were increased with increasing *N* to yield about 1000 hits inside the signal set.

The volume *V_x_* is evaluated numerically as in the low-pass case while the *N*-cube with its volume of 2*^N^* is replaced by the body D confining the signal set, the volume of which is $\;V_{D}=\pi^{N}$. Equation (7) is replaced by $V_{x}=p_{c}\cdot {\left(\pi \sqrt{P}\right)}^{N}$ accordingly. The 4*σ* confidence interval is calculated by the same procedure as in the low-pass case. The results are presented in Figure 10.

The estimated power factor is *γ* = 0.2907, identical to that in [18]; here, we were able to evaluate the 4*σ* confidence interval, which is less than 1% of the power factor.

### 4.3. Upper Bound

The upper bound in the band-pass case is evaluated similarly to the low-pass case. Both are evaluated in spaces of real numbers. The number of dimensions rises from *N* to *2N* and the noise power in each real dimension is reduced by a factor of 2. This results in Corollary 4.

**Corollary** **4.** *In the band-pass case,*

(29) $$\;h\left(y\right)\leq log(V_{w})$$

*where V_w_ is volume of W and W is the Minkowski addition* $W=X+R_{f}\cdot B.\;B$* is an 2N-ball of unit radius. R_f_ is*

(30) $$\;R_{f}=\sigma_{n}\cdot \sqrt{e}\;{\left[\Gamma (N+1)\right]}^{1/\left(2N\right)},$$

*the approximate value is* $R_{f}\approx \sigma_{n}\cdot \sqrt{N}$.

**Proof** . Note that the proof of Theorem 2 was independent of the shape of X. The difference in Corollary 4, relative to Theorem 2, is just replacing σ and *N* by $\sigma /\sqrt{2}$ and *2N*, respectively. The approximate expression of *R_f_* follows immediately. The exact expression (30) follows by inserting $\sigma /\sqrt{2}$ and *2N* into (23). □

The numerical evaluation of the upper bound closely follows the low-pass case described above. The capacity of our continuous time channel is upper-bounded if the peak constraint is only imposed on the Nyquist-rate samples. The bounds on such a channel are presented in [17]. For simplicity, we present a simple EPI-based lower bound on the capacity of such a channel, Equation (38) in [15], which is

(31) $$C_{d}\geq \log_{2}\left(\frac{SNR}{e}+1\right)$$

**Figure 11:**
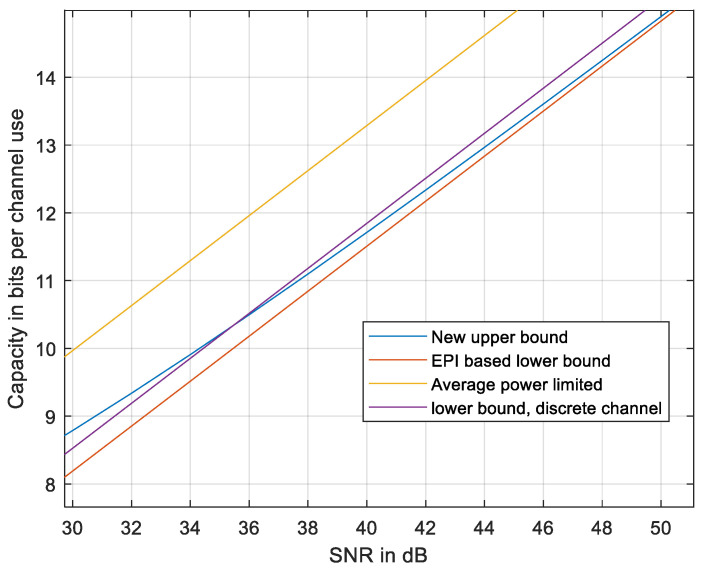
The new upper bound compared to the EPI-based lower bound [18], to the average power-limited capacity, and to the discrete-sampled lower bound using (31). *N* = 51.

It is close to capacity at a high SNR and is, of course, lower than any upper bound. The results are presented in Figure 11.

**Figure 12:**
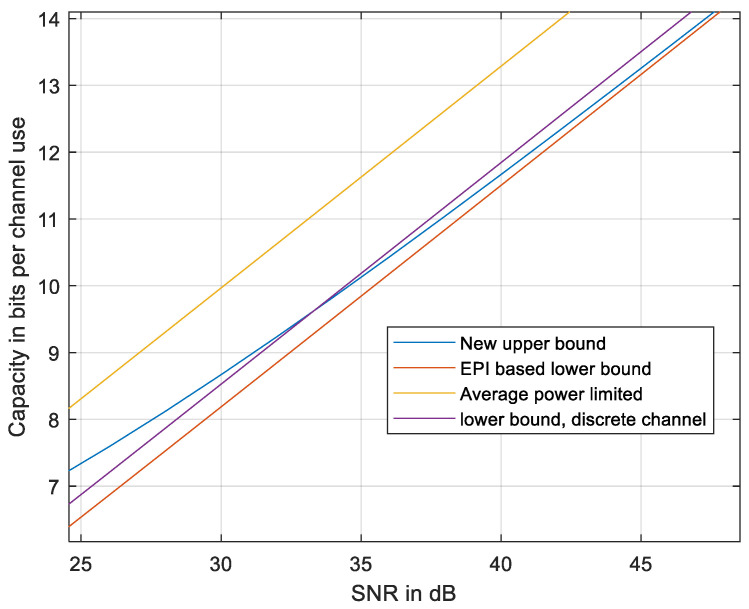
The new upper bound compared to the EPI-based lower bound [18], to the average power-limited capacity, and to the discrete-sampled lower bound using (31). *N* = 31.

As evident from Figure 11, the new upper bound is tighter at high enough SNR, relative to any upper bound based on the discrete-time channel. At even higher SNRs, the new upper bound meets the lower EPI-based bound (28). The bound is slightly nearer to the EPI-based lower bound at reduced *N* (see Figure 12).

The method was verified by applying the peak limit on the Nyquist-rate samples only with results qualitatively similar to those in the low-pass case in Figure 9.

## 5. Conclusions

The important problem of the capacity of Peak-Power Limited and Band Limited (PPBL) signals over the AWGN channel was investigated. We present Theorem 2 upper-bounding the entropy of the received signal leading to an upper bound on capacity; the upper bound is geometry-based and dependent on the shape of the signal set, not only on its volume. We computed an upper bound on capacity applicable to Cyclic Prefix-assisted Frequency Domain Equalization (CP-FDE) and OFDM-type signals. At a high SNR, the new bound is tighter than the previously known upper bounds and approaches the lower bound derived in previous work. We found that, at a high SNR, the change from average to peak power constraints with the same power parameters incurs a cost of about 7.5 dB in the low-pass case and about 5.4 dB in the band-pass case, as reflected by the power factor γ. The previous upper bounds on γ were 2/πe in [9] in the low-pass case and 1/e in [15] and in (31) in the band-pass case, corresponding to a smaller power cost of 6.3 dB and 4.34 dB, respectively. We verified the previous lower bound using a simpler numerical method and established the accuracy of the result. Previous bounds based on the volume of the transmitted signal set yielded EPI-based lower bounds, e.g., [18]. Here, we utilize the effective volume of the received signal set, which is influenced by the volume and the shape of the transmit signal set to derive an upper bound.

Future directions: The upper-bounding technique might be improved and the bound may be tightened, e.g., by more detailed exploitation of the geometry of the problem. The same geometry-based bounding technique should be applicable over the AWGN channel to other signal sets which are convex, such as some cases of MIMO, e.g., [26], the constrained Gaussian channel [20] and PPBL frequency-selective channels. The effort to find effective and practical modulation and coding techniques, e.g., [11,12], should be continued.

## Data Availability

Data is contained within the article and its figures.

## Appendix A. Reshaping the Sections

We presented above a partitioning of the region of the part of the received signal set X, exterior to the transmit signal set Y, into sections. The shape of section *i* is defined by its *S_i_*(*g*)*,* its contribution to *h*(***y***) is denoted *h_i_*, and *p_i_* is the probability that ***y*** is in the section. Each section is associated with its equivalent section having the same *h_i_* and *p_i_*, having the same *S_i_*(*g*) up to *g = R_f_*, and a volume $V_{i}^{\prime }$ over which ***y*** is distributed uniformly. We presented conical-shaped and cylindrical-shaped sections. To ensure that the partition of the whole Y exterior to X into the sections is possible, we show below that the section types can be reshaped to any desired form and produce all of the shapes needed for the partition, with no need to increase *R_f_*. As explained above, *S_i_*(*g*) is non-decreasing with increasing *g* before and after the reshaping.

The first type of reshaping involves the region *g > R_f_*. We shall reshape a section characterized by *S*_1_(*g*) into a section characterized by *S*_2_(*g*) by moving some mass of *S*_1_(*g*) from the higher to the lower region of *g*. We derive *S*_2_(*g*) from *S*_1_(*g*) by increasing *S*_1_(*g*) by *S_d_* in a narrow region of *g*, with a width *dg* around *g*_1_, and decreasing it by $S_{d}\frac{p_{y}\left(g_{1}\right)}{p_{y}\left(g_{2}\right)}$ around a narrow region of *g* around *g*_2_, such that *g*_1_ < *g*_2_; this keeps *p_i_* in (19) constant. There are two cases: Case 1 is *R_f_ < g*_1_ *< g*_2_. In this case, *h_i_* in (18) decreases by

(32) $$p_{y}(g_{1})S_{d}\log\frac{p_{y}\left(g_{1}\right)}{p_{y}\left(g_{2}\right)}dg$$

and so the *R_f_* of the equivalent section can be reduced, as evidenced in (22). Case 2 is *g*_1_ *< R_f_ < g*_2_. As before, *h_i_* decreases and, additionally, the increase in *S*(*g*_1_) increases the volume of the section. Then *R_f_* decreases by both of these two factors.

The second type of reshaping is adding a volume element in the region *g < R_f_*. This is performed in the following steps: First, *S_i_*(*g*) is increased anywhere in the region *g < R_f_* of the original set, adding volume to it and increasing its *h_i_* and its *p_i_* as, governed by (17), (18), and (19). Then *S_i_*(*g*) is modified identically in the equivalent section, but using the same *p_y_*(*g*) in the added region as in the original section. At this stage, the changes in the equivalent section of the volume, the shape *S_i_*(*g*), entropy *h_i_*, and probability *p_i_*, are identical to those in the original section; thus, *R_f_* does not change. Finally, the PDF of ***y*** in the equivalent section is uniformized, keeping *p_i_* constant; this final step does not decrease *h_i_* in the equivalent section, enabling us to keep the original *R_f_* or reduce it.
